# Supplementary material for: Small group size promotes more egalitarian societies as modeled by the hawk-dove game
Source: PLoS One. 2022 Dec 27;17(12):e0279545. doi: 10.1371/journal.pone.0279545 (PMC9794044; doi:10.1371/journal.pone.0279545)
Supplement: Attachment — Submitted filename: small.docx [file pone.0279545.s001.docx]

Small group size promotes more egalitarian societies as modeled by the hawk-dove game

The authors investigated the effects of group size based on Hawk-Dove games, trying to understand the organization in primate social systems. It is very interesting. However, the manuscript (MS) needs to improved in following aspects before I could recommend it to publish in Plos one.

#1. Authors should explain the reason of fixed parameters in table 1.

#2. In section 3.1, authors introduces Hawk-Dove game, as well as the payoff matrix in Fig. 1. However, the important parameters b and c are better to explain in detail in section 3.1 for readers to get a clear image of the game model.

#3. In Fig. 3, authors should set the color bar style as follows: the lower value of in the behind and higher value in the front position. And the color bar are too small to see clearly on the whole.

#4. In Fig. 4, the color bar style are obscure. Author could try to make them bold or bigger to see clearly.

#5. There is too short in main text of fig.4. Are there another aspects to show the effects of group size?
#6. The legends of figures are also simple. Authors may improve their current MS if the figure captions would be made more self-contained. More precisely, what panels are shown for which parameter values, one could also consider a sentence or two saying what is the central theme or message of each figure.

#7. In model section, agents’ offspring either disperses to another randomly selected group in the population with probability d, which is more like the mobility of the agent. Is the parameter d is defined as natal dispersal rate? However, it is very interesting if the authors could discuss the results with the following work for Fig. 5: Li et al.; Applied Mathematics and Computation, 435 (2022) 127456;

#8. Authors investigate the effects of group size theoretically. The work: Jiang et al.; Applied Mathematics and Computation 410 (2021) 126445 explored it experimentally. The section of results could be improved by discussing them.

#9. The authors should check the writing and expression carefully, for example, the referee [22] should be “[Nature](https://www.nature.com/), 428, (2004) 643–646”. There is a long blank space between line 145 and line 146.
